# Investigating the roots of successful IT adoption processes - an empirical study exploring the shared awareness-knowledge of Directors of Nursing and Chief Information Officers

**DOI:** 10.1186/s12911-016-0244-0

**Published:** 2016-01-27

**Authors:** JD. Liebe, J. Hüsers, U. Hübner

**Affiliations:** Department of Business Management and Social Sciences, Health Informatics Research Group, Hochschule Osnabrück, P.O. Box 1940, Osnabrück, D-49009 Germany

**Keywords:** IT adoption, IT diffusion, Awareness-knowledge, IT stakeholder, Chief information Officer, Director of nursing

## Abstract

**Background:**

The majority of health IT adoption research focuses on the later stages of the IT adoption process: namely on the implementation phase. The first stage, however, which is defined as the knowledge-stage, remains widely unobserved. Following Rogers’ Diffusion of Innovation Theory (DOI) this paper presents a research framework to examine the possible lack of shared IT awareness-knowledge, i.e. an information gradient, of two crucial stakeholders, the Chief Information Officer (CIO) and the Director of Nursing (DoN). This study shall answer the following research questions: (1.) Does this gradient exist? (2.) Which direction does it have? (3.) Are certain health IT (HIT) attributes associated with a potential gradient? (4.) Which determinants of diffusion go along with this gradient?

**Method:**

Results of two surveys that focused on the topic “IT support of clinical workflows” from the viewpoint of CIOs and DoNs with corresponding datasets from 75 hospitals were used in a secondary data analysis. The gradient was operationalised by measuring the disagreement of CIOs and DoNs on the availability and implementation status of 29 IT functions. HIT attributes tested were relevance and market penetration of the IT functions, determinants of diffusion were inter-professional leadership and IT service density.

**Results:**

The analysis revealed a significant disagreement on the availability of 9 out of 29 HIT functions. In 23 HIT functions, the CIOs reported a higher implementation status than the DoNs, which pointed to a trend for a unidirectional gradient. The disagreement was significantly lower when the relevance of the IT function was high. Both determinants of diffusion correlated significantly negative with the degree of disagreement.

**Conclusion:**

This is the first study to empirically examine shared awareness-knowledge of two IT-stakeholders that are crucial for triggering IT adoption on the frontline level in hospitals. It could be shown that a gradient and thus a lack of shared awareness-knowledge existed and was associated with certain factors. In conclusion, hospitals should implement improved cooperation between IT staff and clinicians and IT service density when establishing the prerequisites for successful IT adoption processes.

## Background

### Awareness-knowledge as a blind spot in IT adoption research in healthcare

IT adoption in healthcare has been studied increasingly in the recent decade [1–7]. Different concepts regarding the process of adopting health information technology (HIT) have been applied (e.g. deployment, assimilation, implementation, routinisation) [1, 4, 5, 8, 9]. Most of them are inspired and guided by Rogers’ Diffusion of Innovationin^1^ Theory (DOI). The adoption process for individuals is traditionally presented in five stages: awareness, persuasion, decision, implementation, and confirmation [9]. Even though this linear model can be applied for complex healthcare organisations [10, 11], there is strong evidence that organisations move back and forth between the adoption stages and thus follow a recursive path during adoption [1, 8]. Furthermore, there are indications that different adoption processes exist on different levels of organisational decision-making (strategic, operational and frontline^2^) [12]. The strategic level is regarded most crucial for the successful adoption of HIT during the pre-implementation stage, i.e. the decision for the right investment in HIT that is aligned with strategic goals [11–15]. In later stages successful adoption of HIT depends more strongly on processes at the operational and frontline level, IT champions who are enthusiastic about the innovation [16], education and training of the bedside nurses [17], relative advantage [18], and generally on the fit between individual users, technology and clinical tasks and processes [12, 14, 19]. These factors are well-known.

Even though these findings are crucial for understanding how HIT innovations become finally accepted and used, most studies bypass a fundamental stage of IT adoption on higher levels of organisational decision-making: the knowledge-stage. The knowledge-stage is of particular interest in situations if the adoption process is not supported by legal regulations of the government^3^.

Rogers (2003) divides this stage into three separate knowledge types: awareness-knowledge, how-to-knowledge and principle-knowledge. Awareness-knowledge occurs when an adoption unit becomes aware of an innovation’s existence. The other two knowledge types contain information on how to use the innovation and how and why it works [9]. Among the three knowledge types, awareness-knowledge of major stakeholders is the bottleneck of the adoption process, in particular in complex organisations. Only if key decision makers gain and share awareness-knowledge about HIT innovations, adoption can proceed in either linear or recursive processes.

Awareness-knowledge, as part of Rogers’ model of the adoption-decision process, is theoretically associated with three main components of the DOI theory: the adoption-unit, the diffusion and the innovation [20]. In order to investigate how awareness-knowledge is acquired and shared in healthcare organisations it is therefore important (1) to identify the units of adoption whose awareness-knowledge is most crucial, (2) to determine key attributes of HIT innovations which facilitate awareness-knowledge and (3) to find determinants of diffusion which particularly affect the acquisition and sharing of awareness-knowledge.

### Awareness-knowledge of crucial decision making units

Adoption research very often focused on simple innovations for which the unit of adoption is the individual and adoption occurs by simple imitation [8, 9]. In complex healthcare organisations the adoption-decision by individuals on the frontline-level is rarely independent of other decisions so that the unit of adoption is rather a team, a group of professionals, a department or an entire organisation [8]. Therefore representatives of these social networks, who typically work on the strategic or operational level, will execute the actual adoption-decision for HIT innovations [11, 13, 14]. Only if these decision-making units (DMU) [9, 21] gain awareness-knowledge they will initiate and lead the adoption processes on the frontline level [22, 23]. The way these decisions are executed is highly influenced by the hierarchy of the networks and may be either contingent (dependent on decisions made by someone else), collective (the individual has a “vote” but ultimately must acquiesce to the decision of the group) or authoritative (the individual is simply told whether or not to adopt it) [8, 9].

As HIT innovations require technical and clinical knowledge to be adopted successfully [1] clinical as well as IT professionals are crucial DMUs on the strategic and operational level [14, 17]. Several studies provide evidence for the positive effect of the involvement of clinical leaders in the process of HIT innovation adoption (e.g. [12]). Geibert for example stated that nurses are more often involved in the early stages while physicians join during later phases [21], which proves the crucial role of nurses represented by the directors of nursing (DoN). The chief information officer (CIO) acts ideally both on the strategic level, i.e. to align organisational strategies with technical solutions [24], as well as on the operational level, i.e. to support the practical realisation of the IT concepts.

### Attributes of HIT innovations that facilitate the creation of awareness-knowledge

Many studies support the notion of key attributes of innovations explaining a great amount of the variance in their adoption rates [8]. Standard attributes that are often cited are relative advantage, compatibility, complexity, trialability and observability [8, 21, 25].

Another attribute of HIT innovations, which might facilitate the acquisition of awareness-knowledge, is task issue [8], i.e. the relevance of the innovation for the adopting group to perform certain tasks. Although the question is still not answered whether need follows awareness or the other way around [9], there is evidence that the relevance for the potential adoption-unit, e.g. group of professionals or department, and furthermore the fit between the technology, the adoption-unit and the clinical tasks facilitates adoption [7, 19], which includes the acquisition of awareness-knowledge.

The number of adopters of a specific technology itself may have a positive influence on the diffusion of the technology within the community and hereby may become a crucial attribute for the acquisition of awareness-knowledge. The larger the number of adopters is, the better are the opportunities to communicate about the innovation and to become aware of its existence. This phenomenon is reflected by the first half of Rogers’ bell shaped adoption curve [9]. The more adopters there are the higher is the increase in adoption. This trend is attenuated after the point of inflection, i.e. the first 50 % of adopters. From there on the number of adopters obviously only plays an inferior role in adoption.

### Determinants of the diffusion that facilitate the acquisition and sharing of awareness-knowledge

Diffusion is the process by which single adoption-units spread innovations through different communication channels among other members of a social network [9]. There are different cultural and structural determinants that were found to particularly influence the acquisition of awareness-knowledge. Probably the strongest determinant on diffusion is interpersonal influence through social networks, which is defined as the pattern of advice and communication among members of a social network [8, 26]. Collaborative relationships between clinical- and IT professionals on the strategic and operational level were found to help building shared knowledge about HIT innovations [8, 12, 17, 27]. Interactions can be facilitated in informal ways with the help of boundary spanners and champions [25] or in formalised ways through cooperative projects [1, 11, 28, 29].

Another determinant of diffusion that will facilitate the acquisition and sharing of awareness-knowledge of HIT innovation is the network structure within and beyond the adoption-units, especially in multifaceted, highly fragmented healthcare organisations where many different groups use various technologies [1]. Different professional groups have different types of social networks, which influence the diffusion and the way awareness-knowledge is cascaded through the organisation. Whereas physicians tend to operate in informal, horizontal networks, nurses rather have formal, vertical networks [8, 30]. A number of studies found evidence for a strong connection within professional groups and weak across them, which in turn leads to successful diffusion within certain adoption-units but slow diffusion across them (e.g. [31]).

Besides interpersonal influence and network structure the number of IT specialists – relative to the size of the organisation – may also affect the acquisition and sharing of awareness-knowledge. If there are sufficient IT experts available communication between clinicians and IT staff members is easier and allows the clinicians to better access knowledge, new ideas and technical expertise, which then facilitates the adoption of HIT innovations [32, 33].

### Research framework

Shared awareness-knowledge of key DMU on the strategic and operational level is a fundamental stage of HIT innovation adoption in complex healthcare organisations. Only if clinical and IT professionals, who are in the position of key DMUs, gain and share awareness-knowledge they will initiate IT adoption on the frontline level. As the literature had shown different determinants of diffusion and attributes of the HIT innovation can influence the acquisition and sharing of awareness-knowledge. At the same time, a lack of shared awareness-knowledge might become a powerful barrier that counteracts successful IT adoption.

Following these findings we propose a research framework in which we hypothesise that a gradient exists between the awareness-knowledge of technical and clinical key DMUs in complex healthcare organisations and that this gradient is associated with determinants of diffusion and attributes of the HIT innovation.

**Fig. 1:**
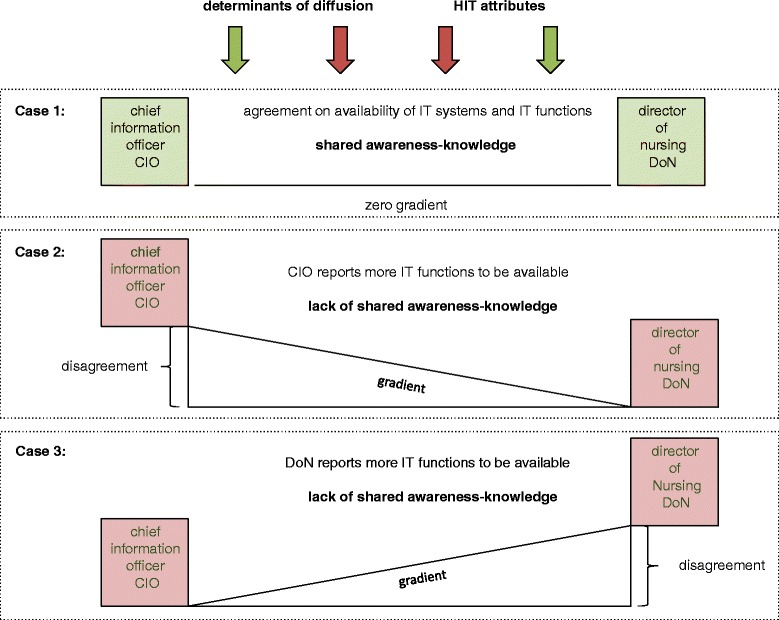
Research Framework

If the gradient is zero there is shared awareness-knowledge, which marks the ideal state (Fig. 1 Case1). If there are differences of awareness-knowledge within the two professions, the key DMUs, the gradient deviates from zero and the gradient deflects to either side (Fig. 1 Cases 2 and 3). Thus agreement between the two groups denotes shared awareness-knowledge, whereas disagreement indicates a lack of shared awareness-knowledge and goes along with either a positive or negative gradient. This system of balance and imbalance is affected by determinants of diffusion and attributes of the HIT system. These factors can act as facilitators or barriers to shared awareness-knowledge (Fig. 1 green and red arrows).

This study shall answer the following research questions:

1. Is there a gradient between the CIOs’ and DoNs’ awareness-knowledge?
2. Is this gradient uniform from CIOs to DoNs respectively vice versa or does the direction of the gradient vary?
3. Are there functions with certain HIT attributes that are associated with a lower or higher gradient?
4. Which determinants of diffusion go along with this gradient and is there an interaction between determinants of diffusion and HIT attributes?

## Methods

### Data

In order to answer the research questions, an already existing dataset, which was captured in two separate surveys, was analysed [34, 35]. Both surveys focused on the topic “IT support of clinical workflows”: the first from the perspective of the CIOs, and the second from the DoNs. The surveys utilised one questionnaire with questions shared by both groups and a section that was specific to each group. The common items covered the IT availability (especially of IT functions). The specific section included structural and managerial determinants. The questionnaires were made available online utilising Unipark and sent to 1.317 CIOs and 1.754 DoNs in German hospitals in 2013 via e-mail. These persons had been identified as CIOs or other persons in charge of IT and as directors of nursing in a manual search based on all 1996 hospitals in Germany [36]. The response rate for the CIO survey was 19.7 % (*n* = 259) and for the DoN survey 26.5 % (*n* = 464). Two variables, the number of nursing staff and the number of organisational units were collected via secondary analyses. Table 1 presents the items that were considered in this study.

**Table 1:** Type, number, examples, and response categories of the items shared by both groups

Type of item	Number of items	Example	Response categories
IT functions	29	Is there a system for clinical reminders in your organisation?	-available in at least one unit -implementation^a^ started -no implementation -no response/I don’t know
Inter-professional teamwork	3	Is there a combined project-leadership of IT staff and clinicians?	-yes -no
IT service density	1	Ratio of IT employees to nurses	-percentage

^a^In the original questionnaire we asked for systems “fully implemented in all units” and “fully implemented in at least one unit but not in all”. In order to avoid misunderstandings we combined these categories to „fully implemented in at least one unit“

We studied the gradient of awareness-knowledge between CIOs and DoNs by measuring the disagreement over the existence and implementation status of 29 IT functions^4^. These 29 IT functions of a HIT system cover many IT applications in a hospital of different types. They were adapted from the list of functions published by Jha and colleagues [37]. Attributes of IT functions considered were market penetration (low vs. high) and relevance for nursing (nursing-relevant vs. non-nursing-relevant).

Finally we choose inter-professional teamwork and IT service density as determinants of diffusion. Inter-professional teamwork was measured by the categories “combined project-leadership (IT staff and clinicians)” versus “exclusive project-leadership of IT staff” or “exclusive project-leadership of clinicians” and IT service density by the “ratio of IT employees to nurses”.

### Matching of data sets

As both surveys were conducted separately and the questionnaires were anonymised, hospitals in which both professional groups had participated had to be identified and the respective data sets had to be matched. The identification of organisations with the participation of both groups was rule based and followed the scheme that CIOs and DoN had to provide identical answers in three demographic questions, i.e. postcode, ownership, and hospital type whereby the postcode had to be identical before the other two characteristics were checked. The results were inspected for quality and plausibility by three persons independently. Finally, 75 hospitals were identified that met the criteria. Cases with missing values from at least one professional group were generally discarded with regard to this item. In case participants had actively ticked “no response” this answer was counted as a valid value in the sense of “I don’t know”, because it can reveal a lack of information flow in either direction. The category “no response” can be interpreted in three possible ways: (1) retention for providing further information, (2) semantical difficulties in understanding the question and (3) “do not know”. As all participants provided information about the implementation status of at least 10 functions and as the wording of all questions was similar, we assumed that ticking “no response” could not be interpreted as the first two possibilities. Therefore in these cases we interpreted “no response” as “do not know”.

### Analysis of the gradient between CIOs and DoNs

To test for a gradient between the awareness-knowledge of CIOs and DoNs the data were analysed in a stepwise manner starting with a highly condensed parameter, drilling down to the item level and single frequencies (Tab 2). For all analyses, the gradient was operationalised by the strength of disagreement over the implementation status of IT functions reported by CIOs and DoNs.

**Table 2:** Overview of the steps of analysis in relation to the research questions

Research questions	Steps of analysis
Is there a gradient between the CIOs’ and DoNs’ awareness-knowledge?	(1.) Comparison of group-means for the reported number of available IT functions regarding the CIO and the DoNs using a paired *t*-test.
	(2.) Comparison of group-means/ranks between the two professional groups on the level of IT functions using the Wilcoxon-test.
	(3.) Visualisation of the relative strength of disagreement via contingently tables for each IT function.
	(4.) Calculation of the relative strength of the non-directional disagreement for each IT function.
Is this gradient uniform from CIOs to DoNs respectively vice versa or does this gradient vary?	(5.) Calculation of the direction of disagreement between CIOs and DoNs for each IT function.
	(6.) Calculation of the relative strength of directional disagreement between CIOs and DoNs for each IT function.
Are there certain HIT attributes that are associated with a lower or higher gradient?	(7.) Categorisation of IT functions according to the HIT attributes “market penetration” and “relevance” and calculation of disagreement scores for each type of IT functions.
	(8.) Testing for significant differences of the disagreement scores between different types of IT functions - classified by the relevant HIT attributes - using a *t*-test.
Which determinants of diffusion go along with this gradient and is there an interaction between determinants of diffusion and HIT attributes?	(9.) Computation of correlations between the disagreement scores for all IT functions respectively for each type of IT function and the determinants of diffusion “inter-professional teamwork” and “IT service density”. Correlation between the determinants of diffusion and hospital characteristics.

**Fig. 2:**
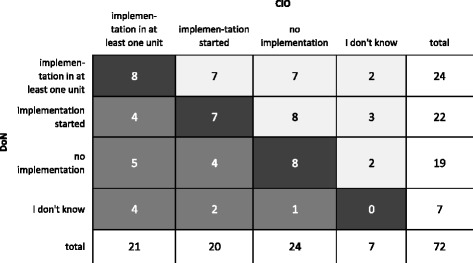
Contingency table of the IT function medical guidelines (n=72)

In a first step, the analyses were based on scores that summarised the number of IT functions reported to be available by the CIO and by DoN. Group-means between the two professional groups were tested for significance by paired t-tests. In a second step, different IT functions were studied separately. The implementation status as judged by the two professional groups was compared individually for each IT function to give a rough impression about the potential differences between the two professions. Group differences were tested for significance using the Wilcoxon-test and alpha was set to 0.05. In order to adjust for alpha inflation through multiple testing, the Bonferroni correction was applied. To further display the strength of disagreement the judgments of the CIOs and DoN were visualised in contingency tables (one per IT function) (Fig. 2). The judgments of the CIOs were placed in the horizontal direction and those of the DoNs in the vertical direction. Therefore, the lower triangular matrix (Fig. 2 dark grey cells) displayed the frequencies of judgments where the CIOs reported a higher implementation status and the upper triangular matrix (Fig. 2 light grey cells) displayed the frequencies of judgments where the DoNs reported a higher implementation status. The relative strength of the total disagreement (non-directional disagreement) per IT function was calculated by adding the frequencies of the lower triangular matrix and these of the upper triangular matrix and by dividing this sum by the number of answers.

### Analysis of the direction of the gradient

Information about the direction of disagreement was gained from computing the difference between the lower and the upper triangular matrix per IT function. A negative value indicated a higher implementation status reported by the CIOs, whereas a positive value indicated a higher implementation status reported by the DoNs. The sign and the magnitude of the difference divided by the number of answers presented the relative strength in which the disagreement tended into one direction (directional disagreement).

### Analysis of the gradient with regard to HIT attributes

Four scores were calculated to test if the disagreement differentiates between certain groups of functions, i.e. a disagreement score for functions with (1.) a low market penetration and (2.) a high market penetration, as well as for (3.) nursing-related and (4.) non-nursing-related functions. The classification of functions with different levels of market penetration followed Rogers’ innovation adoption curve [9]. Functions that where implemented in less than 50.0 % of the German hospitals (equivalent to the Rogers’ groups: innovators, early adopters, early majority) belonged to the category “low penetration”, and all other functions fell in the category “high penetration” (equivalent to the Rogers’ groups: late majority and laggards) [34]^5^.

- *functions with low market penetration* comprehended the nursing documentation, medication loop, intensive care record, medical guidelines, clinical reminders, clinical alerts, decision support drug therapy, drug administration record, pharmacy, patient identification, critical incidents reporting system, electronic archive, health information exchange.
- *functions with high market penetration* were the medical summary, minimum medical data set, surgery record, anaesthesia record, order entry laboratory, order entry radiology with images, order entry radiology without images, order entry electrophysiology, specimen identification, materials management, medication order entry, meal ordering, inpatient management, outpatient management.

Six experts (three scientists in nursing informatics and three scientists in medical informatics) classified the 29 IT functions into nursing-related and non-nursing-related functions.

- *Nursing-related functions* comprised the nursing documentation, intensive care record, order entry laboratory, clinical reminders, clinical alerts, specimen identification, drug administration record, surgery record, anaesthesia record, patient identification, critical incidents reporting system, materials management, medication order entry, meal ordering, inpatient management, outpatient management, health information exchange.
- *Non-nursing-related functions* included the medical summary, minimum medical data set, medication loop, decision support drug therapy, pharmacy, order entry radiology with images, order entry radiology without images, order entry electrophysiology, medical guidelines, electronic archive.

Each disagreement score expressed the percentage of disagreement between CIOs and DoNs on the availability of IT functions in their hospital (coded as available or not). If - for example - the CIO and the DoN disagreed on the availability of 8 out of the 17 nursing-related functions, the disagreement score for the regarding hospital amounted to 47,1 %. A paired *t*-test was performed to determine if significant differences between the contrastive pairs existed, e.g. functions with low vs. high market penetration.

### Analysis of the gradient with regard to determinants of diffusion

In order to test if well known determinants of diffusion could be associated with the gradient, three items that described the organisational management and one item that describes the organisational structure were correlated with the disagreement scores for all functions, nursing- and non-nursing-related functions, functions with low and high market penetration and tested for significance with alpha set to 0.05. To operationalise the managerial determinants the following items where used (1) combined project-leadership of IT staff and clinicians (yes/no), (2) exclusive project-leadership of IT staff (yes/no), (3) exclusive project-leadership by clinicians (yes/no). They represent the determinant inter-professional teamwork. The structural determinant was operationalised by the ratio of IT employees to nurses. A potential interaction between HIT attributes and determinants of diffusion was tested by correlating the disagreement scores of IT function with low vs. high market penetration and nursing-related versus non-nursing related IT functions with the managerial and structural determinants, which represented the determinants of diffusion. To test for inter-correlations the determinants of diffusion where correlated with the following hospital characteristics: hospital size (total number of beds), ownership (private vs. non private), type (part of a hospital group versus stand-alone hospital) and teaching-status (academic hospital versus non academic hospital). We computed the point-biserial correlation coefficient for correlating bi-nominal and metric features (e.g. size and exclusive project-leadership of clinicians), computed the Pearson correlation coefficient for the size and ratio of IT employees to nurses and finally cross-tabs and the phi-coefficient for all other combinations.

### Overview of research questions and methods

Table 2 provides an overview of the different steps of analysis and their relation to the research questions.

## Results

### Participating hospitals

The sample contained 75 hospitals of all types of ownership and size. Table 3 gives an overview of the hospitals in this study and their characteristics “ownership” and “size”.

**Table 3:** Ownership and size of hospitals in the sample (*n* = 75)

Hospital demographics	Absolute frequencies	Relative frequencies in %
Ownership: private hospitals	13	17.3 %
Ownership: public hospitals	62	82.7 %
Size: up to 399 beds	46	61.3 %
Size: 400 to 799 beds	19	25.3 %
Size: 800 and more beds	10	13.4 %

### Existence and direction of a gradient

The average number of IT functions available as reported by the CIOs was 17.5 (SD ±5.3). The DoN reported an average of 14.4 (±3.9) IT functions available. The two groups differed by about three IT functions, which was significant in the paired *t*-test (*p* <0.00).

Table 4 shows the result of the Wilcoxon-test and summarised frequencies of the contingency tables (example see Fig. 2). Out of 29 IT functions, there were nine with significant differences in the judgment of the implementation status. Column two presents the relative strength of the non-directional disagreement, which varies between 73.6 % for *clinical reminders* as the highest non-directional disagreement and 16.4 % for *order entry laboratory* as the lowest non-directional disagreement. In total, 14 IT functions showed a non-directional disagreement of 50.0 % and more. Column three presents the relative strength of the directional disagreement. A negative difference was calculated for 23 functions, which indicates a higher implementation status reported by the CIOs. This disagreement showed an absolute value of 20.0 % and more for 13 IT functions. The strongest disagreement where CIOs reported a higher implementation status concerned *order entry radiology without images* (−47.7 %). The strongest disagreement in the other direction was related to *medical guidelines* (+12.4 %). Column four presents the relative frequencies of the upper triangular matrix where the DoNs reported a higher implementation status and column five presents the relative frequencies of the lower triangular matrix where the CIOs reported a higher implementation status.

**Table 4:** Direction and strength of disagreement for the individual IT function sorted by z-values (bold: significant after Bonferroni correction)

		(a)	(b)	(c)	(d)
IT functions for supporting…	Wilcoxon (z-value)	Non-directional relative strength of disagreement (c + d) in %	Direction and relative strength of disagreement (c-d) in %	Sum of relative frequencies upper triangular matrix (DoN) in %	Sum of relative frequencies lower triangular matrix (CIO) in %
Order entry radiology without images (*n* = 67)	−5.1	62.7 %	−47.7 %	7.5 %	55.2 %
Health information exchange (*n* = 71)	−3.8	60.6 %	−32.4 %	14.1 %	46.5 %
Outpatient management (*n* = 73)	−3.5	34.2 %	−23.4 %	5.4 %	28.8 %
Inpatient management (*n* = 72)	−3.5	23.6 %	−20.8 %	1.4 %	22.2 %
Intensive care record (*n* = 75)	−3.4	54.7 %	−25.3 %	14.7 %	40.0 %
Specimen identification (*n* = 70)	−3.4	41.4 %	−21.4 %	10.0 %	31.4 %
Order entry electrophysiology (*n* = 74)	−3.3	39.2 %	−23.0 %	8.1 %	31.1 %
Order entry radiology with images (*n* = 73)	−3.3	34.2 %	−17.8 %	8.2 %	26.0 %
Nursing documentation (*n* = 75)	−3.2	44.0 %	−22.6 %	10.7 %	33.3 %
Anaesthesia record (*n* = 75)	−3.0	38.7 %	−17.3 %	10.7 %	28.0 %
Minimum medical data set (*n* = 75)	−2.9	30.7 %	−20.1 %	5.3 %	25.4 %
Medication order entry (*n* = 75)	−2.8	46.7 %	−17.3 %	14.7 %	32.0 %
Medical summary (*n* = 75)	−2.7	21.3 %	−15.9 %	2.7 %	18.6 %
Product identification (*n* = 72)	−2.5	65.3 %	−29.1 %	18.1 %	47.2 %
Surgery record (*n* = 55)	−2.5	20.0 %	−16.4 %	1.8 %	18.2 %
Critical incidents reporting system (*n* = 67)	−2.4	61.2 %	−28.4 %	16.4 %	44.8 %
Order entry laboratory (*n* = 73)	−2.4	16.4 %	−8.2 %	4.1 %	12.3 %
Materials management (*n* = 72)	−2.0	37.5 %	−12.5 %	12.5 %	25.0 %
Location identification (*n* = 72)	−1.7	61.1 %	−24.9 %	18.1 %	43.0 %
Patient identification (*n* = 72)	−1.3	54.2 %	−7.0 %	23.6 %	30.6 %
Clinical reminders (*n* = 72)	−1.2	73.6 %	−20.8 %	26.4 %	47.2 %
Decision support drug therapy (*n* = 72)	−0.7	63.9 %	−5.5 %	29.2 %	34.7 %
Pharmacy (*n* = 70)	−0.6	38.6 %	+1.4 %	20.0 %	18.6 %
Medical guidelines (*n* = 72)	−0.6	68.1 %	+12.5 %	40.3 %	27.8 %
Drug administration record (*n* = 72)	−0.5	61.1 %	+2.7 %	31.9 %	29.2 %
Medication loop (*n* = 72)	−0.5	63.9 %	+8.3 %	36.1 %	27.8 %
Clinical alerts (*n* = 72)	−0.3	63.9 %	−2.7 %	30.6 %	33.3 %
Meal ordering (*n* = 72)	−0.2	22.2 %	+2.8 %	12.5 %	9.7 %
Electronic archive (*n* = 72)	−0.2	54.2 %	+4.2 %	29.2 %	25.0 %

### Correlation of HIT attributes with gradient between CIOs and DoNs

All functions were classified by the HIT attributes market penetration {low, high} and relevance {nursing related, non-nursing related}. The relative disagreement for IT functions with a low market penetration was slightly but not significantly higher than for IT functions with a high market penetration (difference of 2.3 % points; *p* > 0.05). For nursing related functions the relative disagreement was significantly lower than for non-nursing related functions (difference of 5.2 % points; *p* < 0.05) (Table 5).

**Table 5:** Group means (± SD) and association of the disagreement scores (in %) for different types of IT functions (*n* = 75)

	Functions with low market penetration: mean (SD)	Functions with high market penetration: mean (SD)	*p*-value
disagreement score	29.9 (±17.9)	27.6 (±16.2)	0.262
	nursing-related functions: mean (SD)	non-nursing-related functions: mean (SD)	*p*-value
disagreement score	28.4 (±14.5)	33.6 (±15.2)	0.02

### Correlation of determinants of diffusion with gradient and interaction between HIT attributes and determinants of diffusion

The correlation between the determinants of diffusion and the overall disagreement score, i.e. for all functions, was significantly positive in case of “exclusive project-leadership of IT staff” and significantly negative in case of “ratio of IT employees to nurses”. “Exclusive project-leadership of IT staff” also correlated significantly positive with “non-nursing-related functions”, whereas there was no other significant correlation of the “ratio of IT employees to nurses” with any other type of IT functions described by HIT attributes. There was a significant negative correlation between “combined project-leadership (IT staff and clinicians)” with the disagreement score for IT functions with a low market penetration showing an interaction between these specific categories of inter-professional teamwork and market penetration. In the majority of the cases the sign of the correlations between the determinants of diffusion and the disagreement scores were identical with the exception of very low correlations, i.e. almost zero correlations. Thus determinants of diffusion were similarly associated with HIT attributes only showing variety in the strength of the correlation. The ratio of IT-employees to nurses correlated positive with private ownership (r = 0.318; *p*-value < 0.05) and with hospital size (r = 0.402; *p*-value < 0.01). No other significant correlations resulted between the determinants of diffusion and hospital characteristics (Table 6).

**Table 6:** Correlation-matrix for disagreement scores and different determinants of diffusion and HIT attributes (bold: sig *p < 0.05; ** *p* < 0.001)

	Determinants of diffusion		Disagreement scores for different HIT attributes			
			Market penetration		Relevance	
		All functions	Low	High	Nursing-relevant	Non-nursing-relevant
Inter-professional teamwork	combined project-leadership (IT staff and clinicians)	−0.232	−0.252*	+0.009	−0.075	−0.214
	exclusive project-leadership of IT staff	+0.344**	+0.252	+0.192	+0.166	+0.357**
	exclusive project-leadership of clinicians	−0.088	+0.091	−0.163	−0.092	−0,127
IT service density	ratio of IT employees to nurses	−0.287*	−0.140	−0.212	−0.154	−0.225

## Discussion

This study is based on the notion that recent IT adoption research in healthcare does not sufficiently consider the existence of shared awareness-knowledge of key decision makers in healthcare organisations. This finding is surprising as shared awareness-knowledge is the origin of the adoption process on the operative and frontline level of those organisations. Our analysis followed the hypothesis that a gradient between crucial stakeholders in the clinical and technical setting existed, and that this gradient appeared as a gap between the awareness-knowledge of the technical and clinical stakeholders. This study focuses on the DoN and on the CIO as they play a significant role at the beginning of the organisational IT adoption once the investment had been decided.

We matched two already existing datasets, which resulted in a sample of 75 hospitals of different size and ownership. For the purpose of this study, we used the responses of the CIOs and DoNs about the implementation status and availability of 29 IT functions.

The first research question investigated the fact if there existed a gradient between the CIOs and the DoNs with regard to awareness-knowledge. We found a significant disagreement between CIOs and DoNs concerning the number of IT functions available in their hospital. This result was also confirmed on the level of individual functions: CIOs and DoNs significantly disagreed on the implementation status of nine functions. Fourteen IT functions showed a total (non-directional) disagreement of 50 % and more. In fact we found just one IT function (*order entry laboratory*) were the total disagreement between CIOs and DoN amounted to less than 20 %. In conclusion to this research question, this study confirms the existence of a gradient.

The second research question asked if this gradient was uniform from CIOs to DoNs respectively vice versa or if the direction of the gradient varied. The comparison of the group means of the total number of IT functions revealed that the CIOs reported significantly more functions to be available than the DoNs. These results could be replicated on the level of individual IT functions: the CIOs reported a higher implementation status for 23 out of the 29 functions. We found the strongest directional difference for *order entry radiology without images* (−47.7 %). Although there were IT functions for which the DoNs reported a higher implementation status than the CIOs, they were rather small with the exception of the IT function *medical guidelines*. The large majority of IT functions yielded a different picture and pointed to a trend for a uniform gradient.

These results are not surprising as the CIOs were responsible for making these IT functions technically available. Yet they also confirm the assumption that technical availability does not automatically result into awareness on the side of the users, in particular in case of software functions, which are sometimes hidden in a complex user interface and become only obvious if the users are explicitly made aware of them. The interpretation of DoNs not being interested in IT and therefore not knowing the details seems rather unlikely because the DoNs in our study participated in the survey by their own choice. The survey itself clearly addressed technical issues right from the beginning and could have been rejected immediately if no interest existed.

We also asked (third research question) whether there were functions with certain HIT attributes that were associated with a lower or higher gradient, i.e. a weaker or stronger disagreement. Examining the results of the individual types of IT functions, with low market penetration (e.g. *health information exchange* or *critical incidence reporting*) seemed to be more vulnerable for a strong directional disagreement than functions with a high market penetration, i.e. CIOs reported a higher implementation status than DoNs. On the other hand, nursing-related functions (e.g. *patient identification*) seemed to show a lower disagreement. To test for a systematic difference we calculated disagreement scores for four groups of IT functions representing the two different HIT attributes “market penetration” and “relevance” and compared the group means of the two contrasting pairs high versus low penetration and nursing-related versus non-nursing related functions. We found a higher disagreement over IT functions with a low than over those with a high penetration although this difference was not significant. For nursing-related functions the disagreement was significantly lower than for non-nursing-related functions. These results indicate that the kind of technology, i.e. the HIT attributes, can be a potential barrier for shared awareness-knowledge – as in the case of the market penetration of the IT function – or it can become a facilitator as in case of the relevance of the IT function. In particular the relevance of the technology for the daily work seems to be associated with a lower gradient. These results correlate with earlier studies, which found technologies to be easier adopted if they were relevant to the performance of the intended user’s work [38].

The fourth and final research question was if determinants of diffusion went along with a lower gradient and if there was an interaction between determinants of diffusion and HIT attributes. We therefore computed correlations between two determinants (inter-professional teamwork and IT service density) and the disagreement between CIOs and DoNs. We hypothesised that inter-professional teamwork, i.e. combined project-leadership of IT staff and clinicians, and higher IT service density, i.e. higher ratios of IT employees to nurses, facilitated shared awareness-knowledge and thus went along with less disagreement on the availability of IT functions. Our results support this assumption. If hospitals exercise a combined project-leadership the disagreement between CIOs and DoN on the availability of IT functions with a low market penetration is significantly lower. This finding is supported by other studies, which found inter-professional teamwork and the involvement of clinicians in management networks to foster adoption especially of technologies, which are new on the market, as these conditions can enable “the development of shared meanings and values in relation to the innovation” ([8], p. 606). In contrast to these results, “exclusive project-leadership of IT staff” correlated significantly positive with disagreement on the availability on all IT functions and on non-nursing related IT functions. This indicates the lack of user involvement. Regarding the determinants of diffusion, we further hypothesised that shared awareness-knowledge depended on service density, which was operationalised by the ratio of IT employees to nurses. As expected, hospitals with a low ratio of IT employees to nurses tended to have higher disagreement scores and vice versa. These results correspond with prior studies (e.g. [39]), which indicated that the number of IT employees seems to be crucial not only for later stages of the IT adoption process but also for the awareness-knowledge phase.

**Fig. 3:**
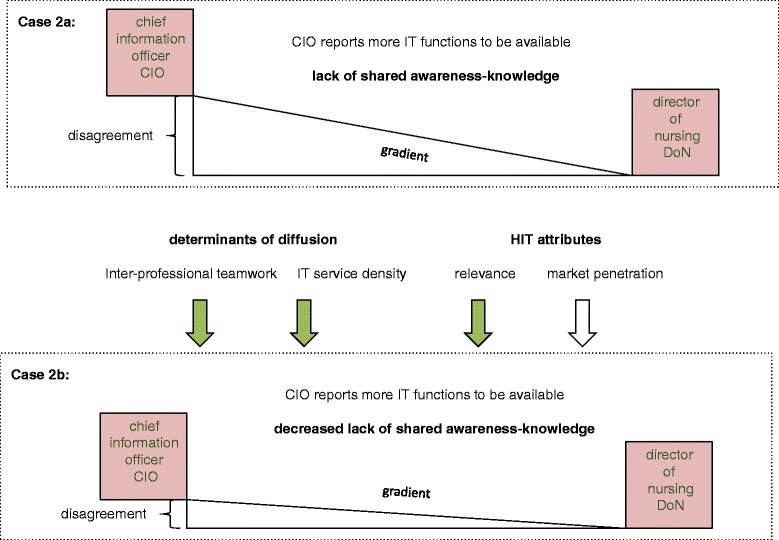
Summaries of the results in context of the research framework

Figure 3 summarises the results in the context of the research framework. It shows the gradient from the CIOs to the DoNs (case 2a), which can be mitigated by inter-professional teamwork and IT service density and by the relevance of the IT functions (case 2b). Market penetration does not seem to play a significant role.

## Limitations

The analysis included 75 hospitals, which is a rather small sample even though it represented hospitals of all size categories and types of ownership.

When studying determinants of diffusion and HIT attributes as facilitators or barriers, it is desirable to draw conclusions in terms of the influence of these factors or the mechanism of action. We, however, computed correlations, which do not give proof of any influence but only of co-existence. The correlations found were small and thus the results need to be replicated and more determinants of diffusion (e.g. strategic alignment, user-training) and more HIT attributes (e.g. software versus hardware) should be tested.

The ultimate goal is to develop a comprehensive model for understanding potential associations and influences. However, the chance to obtain a stable multiple regression model based on *n* = 75 is rather low. Computing simple linear regressions does not provide much added value because the beta coefficients equal the Pearson correlation coefficients. As the correlations are not very high the amount of variance clarified by simple linear regression models would be even lower. This shows that simple regression models cannot explain much variance in this case. Given the value of multiple regression models it is advisable to prepare the work for building such models by exploring further factors that might correlate with well-known determinants of diffusion. We could identity two such factors, namely size and ownership.

We focused on shared awareness-knowledge respectively on the lack of it and we studied some determinants of diffusion. What we did not measure explicitly is interpersonal communication, a powerful driver of shared awareness-knowledge. In our study setting, interpersonal communication served as a latent variable, which might be influenced by inter-professional teamwork and IT service density, but was not measured.

Future studies should investigate the transition from awareness-knowledge to frontline usage, the next step along the IT adoption process. Besides the perspective of CIOs and DoNs, future research could also analyse shared awareness-knowledge of other professional groups, e.g. between CIOs and medical directors.

## Conclusion

This is the first study to empirically examine awareness-knowledge, particularly shared awareness- knowledge of two stakeholders that are crucial for triggering IT adoption on the frontline level in an organisation. The study proposes a research framework and investigates whether there is a hypothesised gradient between the two stakeholder groups and thus a gradient of awareness- knowledge. It also looks at factors, namely determinants of diffusion and HIT attributes that may be associated with this gradient. We identified facilitators and barriers of awareness-knowledge: Low IT service density and exclusive IT staff leadership in IT projects seem to impede the development of shared awareness-knowledge and to build up a gradient. In contrast, combined leadership in IT projects seems to facilitate shared awareness-knowledge and mitigate the gradient. None of these determinants of diffusion was significantly associated with the awareness-knowledge of functions with a high market penetration. In contrast, shared awareness-knowledge on IT functions with a low penetration can benefit from combined IT project-leadership, thus from inter-professional teamwork. It can be concluded that awareness-knowledge of non-IT stakeholders must not be taken for granted. It must be constructed and continually negotiated among all relevant groups. In hierarchical organisations, such as hospitals, shared awareness-knowledge of CIOs and DoNs is the gateway to adoption. Otherwise, often discussed determinants for successful IT adoption might not become effective. This should be taken into account in future IT adoption research. The practical conclusion is that hospitals should establish a combined leadership of IT experts and clinicians in IT projects and should raise the IT service density when establishing the prerequisites for successful IT adoption processes.

## Footnotes

1 According to Rogers’ DOI theory innovations are defined as relative with regard to the adoption unit [9], i.e. a product is defined as innovative if it is new for the adopter.

2 Other authors, e.g. Winter and colleagues [28] distinguish between strategic, tactical and operational levels, which correspond roughly with the above classification.

3 Studies could show that legal regulations are strong drivers of IT adoption [6] because these regulations are announced publicly and are on the mind of the decision makers.

4 These IT functions were studied irrespectively of the overall technical architecture they were embedded in. This means that in some hospitals these IT functions could have been integrated into an electronic health record system in others not. We refrained from targeting architectural issues because we could not expect Directors of Nursing to be familiar with these terms.

5 Product identification and location identification were not considered for the disagreement scores due to missing values.
